# Ethnic-specific discrimination: focus group findings of Korean American emerging adults

**DOI:** 10.3389/fsoc.2025.1658624

**Published:** 2026-01-07

**Authors:** Hans Oh, Woo Jung Amy Lee, Ronna Bañada, Brenda Goh, Bo-Kyung Elizabeth Kim, Yuri Jang, Jimi Huh, Jungeun Olivia Lee

**Affiliations:** 1 School of Social Work, University of Southern California, Los Angeles, CA, United States; 2 University of California, Berkeley, Berkeley, CA, United States; 3 Fielding School of Public Health, University of California, Los Angeles, Los Angeles, CA, United States; ^4^Ewha Womans University, Seoul, Republic of Korea

**Keywords:** racism, discrimination, Asian American, Korean American, emerging adults

## Abstract

**Introduction:**

Disaggregating the Asian American racial category is crucial to understanding ethnic differences in discrimination. However, few studies have qualitatively explored perceptions of whether ethnic discrimination differs from racial discrimination.

**Methods:**

We conducted three focus groups with Korean American emerging adults (*N* = 13) to explore perceptions of racial discrimination. After COVID-19 pandemic and the Black Lives Matter protests, we conducted a follow-up focus group. We combined these findings with collective auto and insider ethnography.

**Results:**

Participants described being stereotyped and conflated with other Asians, with some perceiving positive stereotypes as benign. They identified flaws in existing racism measures. Historic events appeared to heighten awareness of one’s racial and ethnic identity and awareness of systemic racism impacting all people of color.

**Discussion:**

Future studies may seek to revise discrimination measures to better capture ethnic-specific experiences and their implications for health.

## Introduction

Racism is an enduring and intricate system that privileges White people while oppressing people of color in the United States (US) (Bonilla-Silva, 1997; Williams and Mohammed, 2013). Studies have shown that Asian Americans experience racial discrimination on a regular basis (Spencer et al., 2010). Over the past few decades, racial discrimination measures have evolved in order to accommodate the shape-shifting nature of racism, with an on going objective to measure racial discrimination with greater nuance and complexity (Bastos et al., 2010). Along these lines, it is important to interrogate the assumption that the racial discrimination measures used for Asian Americans are adequate for specific Asian American ethnic subgroups, since there is considerable heterogeneity within the Asian American population (Choi et al., 2020).

Historically, early measures of racial discrimination were largely developed based on the experiences of Black Americans. One example of a measure is the Everyday Discrimination Scale (EDS; Williams et al., 1997), which has also been widely administered to Asian Americans (e.g., Yip et al., 2008). While important, the EDS does not resonate uniformly across racial/ethnic groups (Bastos and Harnois, 2020), since Asian Americans have distinct experiences of racial discrimination (Lee, 2005; Spencer et al., 2010). As such, several measures have been developed to center the experiences of Asian Americans, such as the Asian American Racism-Related Stress Inventory (AARRSI) (Liang et al., 2004). The AARRSI was crafted to assess pan-ethnic Asian American racial discrimination, touching on experiences such as language discrimination and positive stereotypes. However, measures like the AARRSI rarely examine experiences that are specific to any particular Asian ethnic subgroup. The assumption has largely been that the broader racial discrimination measures for Asian Americans should sufficiently cover the experiences ethnic subgroups (see Ong et al., 2013).

One qualitative study found that Korean American immigrants and children of immigrants demonstrate a sort of racial ambivalence, neither identifying with “oppressed” minority groups nor the dominant “privileged” white race, resulting in racism denial or minimization (Son, 2014). The denial or minimization of racism may obscure the understanding of racial discrimination experienced by Korean Americans. Additionally, discrimination experiences may be rooted in positive stereotypes such as Asians Americans being good a math (Gupta et al., 2011) or microaggressions where people are impressed that Asian Americans speak English well (Ong et al., 2013). The positive nature and the subtlety may further obscure reports of racism. However, given the sparse research, it is unclear whether such contexts may influence how Korean Americans perceive discrimination.

### Korean American young adults

Another important limitation in the current understanding of racial discrimination is the minimal research that exists on Korean American emerging adults’ experiences. Emerging adulthood (typically aged 18–29) is characterized by exploring one’s identity, reflecting on oneself, but also feeling like one is in a transitional (and somewhat unstable) state, filled with concern and excitement for the future. Emerging adulthood is a critical developmental period for ethnic and racial identity development marked by a deeper reflection, wider exploration, and increased flexibility as well as formation of complex intersection among multiple identities such as social class, political, gender, national identities (Umaña-Taylor et al., 2014). The consolidation of the ethnic and racial identity, defined as a sense of collective identity based on one’s perception of shared heritage, activities, and culture with a particular racial group (Helms, 2003; Yip, 2018), is likely to continue to evolve throughout adulthood and this process is informed by the ecological context in which individual lives are embedded, including the surrounding community (McLean, 2008). During these formative years, ethnic minority young adults may become more aware of racial discrimination experiences, which may also shape their identity trajectories.

The literature on Korean American emerging adult discrimination experience is lacking. While studies have documented the discrimination that Korean Americans face in adulthood, these findings are often not generalizable to emerging adults because of immigration and acculturation differences. Many first-generation Korean immigrants occupy different areas of the labor market (e.g., self-employed, small businesses), and may remain segregated in ethnic enclaves. Emerging adult Korean Americans on the other hand may tend to have more access to educational opportunities and have closer connection to the mainstream society (Min, 2006). Thus the experiences of English-speaking Korean American young adults who spent their childhood in the U.S. may have notably different characteristics compared to the older immigrant generation in how they perceive racial discrimination experiences (Young et al., 2022). Very little qualitative research exists on Korean American emerging adults’ racial discrimination experiences, with a few exceptions including some studies focusing on adopted Korean individuals (Lee et al., 2019; Schires et al., 2020).

Thus, we sought to understand how Korean Americans perceived racial discrimination targeting Asian Americans and whether they perceived ethnic discrimination targeting Korean Americans to be distinct. We embarked on this journey just prior to a complex historical moment that brought issues of race and identity into the forefront of public discourse. In 2020, the COVID-19 pandemic ignited a firestorm of anti-Asian racism, prompting many Asian Americans to either directly experience or witness racial discrimination (Chen et al., 2020; Gover et al., 2020). This moment was also characterized by the Black Lives Matter protests (herein referred to as BLM) following the killing of unarmed Black Americans by the police, which raised awareness of anti-Black racism (Nguyen et al., 2021; Wu et al., 2023). At the same time, Korean popular culture had been enjoying a ‘moment’ internationally during a Korean Wave (i.e., *Hallyu*), with the Korean film *Parasite* winning ‘Best Picture’ at the Academy Awards, and with the Korean pop group BTS winning Billboard’s ‘Top Social Artist’ 5 years in a row from 2017 to 2021 (Kim, 2021). Understanding how these converging historical moments impact emerging adults is critical given that emerging adulthood is a stage when people are still negotiating their identities. Particularly for marginalized groups, historic events (both hostile and affirming) that take place during emerging adulthood may dramatically shape the ethnic and racial identity trajectories (Phinney and Ong, 2007).

We conducted multiple focus groups with Korean American emerging adults in higher education to understand their experiences of racial and ethnic discrimination. We specifically explored their reactions to the AARRSI and whether they felt it captured their experiences of ethnic discrimination. We examined the extent to which the aforementioned historical events might have altered perspectives on racial and ethnic discrimination. We then breached the divide between researcher and participant by employing insider- and auto-ethnography to make sense of the focus group findings and set an agenda for future research.

## Methods

Increasingly, qualitative researchers are recognizing the need to coalesce multiple qualitative approaches to understand complex phenomena, especially that which is emergent and evolving (e.g., Chamberlain et al., 2011; Heath et al., 2018). Thus, we employed a multi-method qualitative approach, consisting of focus groups and insider-ethnography/auto-ethnography to interpret focus group findings and make transparent our own thoughts, reasoning, and sense-making within the context of these historical events. The procedures were approved by the IRB at the University of Sourthern California (UP-19-00248).

### Focus group sample

**Figure 1:**
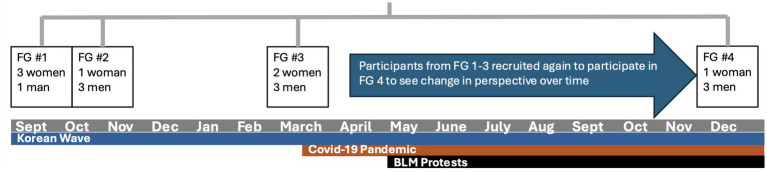
Timeline of focus groups from 2019 to 2020.

We recruited 12 respondents (*N*_p_ = 12) to participate in focus groups between September 2019 and December 2020. We conducted four sequential focus groups comprising Korean American English-speaking emerging adults in higher education and allowed open-ended conversations to organically lead to discovery. Eligibility criteria included individuals who were (1) 18–24 years old and (2) self-identify as Korean American (has lived in the U.S. for at least 5 years regardless of immigrant or visa status). We recruited participants using convenience sampling, drawing from our own social networks through emails and verbal communication. We allowed only four participants in each of the focus groups so that participants would have enough time to share their thoughts on a sensitive topic (Toner, 2009). Upon completion of the initial three focus groups, a set of historical events occurred that we believed would reshape the views of the participants. We leveraged these events to explore the potential changes in perspectives of our focus group participants by re-inviting them for the fourth focus group (*N* = 4). At least one participant from each of the original three focus groups participated in the follow-up. The participants were on average 20 years old and 46% were women. Recruitment flyers were in both English and Korean, but all respondents were English-speaking. All respondents were college educated. We present the size and gender composition of each focus group in Figure 1 (See Supplementary material Table S1A, S1B).

### Procedures

All participants provided informed consent for their participation in the study. Three of the focus groups (pre-COVID) were held in person and the fourth follow-up focus group (during the COVID-19 pandemic) was held via the Zoom online platform. Each focus group lasted approximately 80 min, and each participant received $50 compensation for attending a focus group and an additional $40 for participating in a follow-up focus group. The in-person focus groups were audio recorded and transcribed verbatim through Scribie, a confidential third-party transcription service. The online groups were recorded using Zoom and the transcript was auto-generated and corrected manually by one of the authors. Two researchers coded the data, and used Excel to track all codes and themes. We did not calculate an inter-coder reliability, given the interpretive nature of our inquiry, and that our aim was not to find agreement in seeing the data the exact same way (but see O’Connor and Jofee, 2020 for discussion on this topic).

### Warm-up activities

To prepare the respondents for discussion about racial discrimination, we began each focus group with a warm-up activity that asked participants to list experiences of racial discrimination, and whether the discrimination happened to them personally (direct), to Korean Americans they know (vicarious), or to other Asians whom they do not know personally (group-level). We asked participants to assume multiple vantage points to prime them for the focus group discussion. We then asked participants to complete the AARRSI (Liang et al., 2004).

### Interview questions

Following these two activities, we conducted a semi-structured focus group inviting respondents to reflect on their responses to the AARRSI. Examples of interview questions are available in Supplementary material Table S2. Given the sequential nature of the focus groups, we allowed the interview questions in the fourth focus group to evolve based on emergent findings from prior focus groups. Thus, we were able to invite a subsample of the original respondents to reflect on the recent events related to COVID-19 and whether their perspectives about racial discrimination toward Korean emerging adults have changed since the previous focus group.

### Positionality, insider ethnography, and collective auto-ethnography

This study’s methodology is two-pronged in that our inquiry is informed by two sources, which are the focus group participants, as well as our own lived experiences. As Korean Americans researching our own community, we occupy a unique position from which we can speak to the emergent qualitative findings. We borrow concepts and techniques from the ethnographic literature that often uses terms like “native” or “indigenous” ethnography. We prefer the term “insider-ethnography” when observing the Korean American community and “auto-ethnography” when reflecting on our lived experiences (Boylorn, 2014). We drew from these ethnographic approaches to formulate our own observations as we discussed and made sense of the qualitative findings. Six of the researchers (*N*_r_ = 6) consisting of both faculty and a PhD student bring considerable knowledge to this study having lived in the Korean American community in California with varying generational statuses (1st, 1.5th, and 2nd generation). We wrote memos to document our observations of the Korean American community, as well as our own lived experiences of racial discrimination, and we used our memos in tandem with the focus group data to make our thought processes transparent when interpreting the findings.

**Figure 2:**
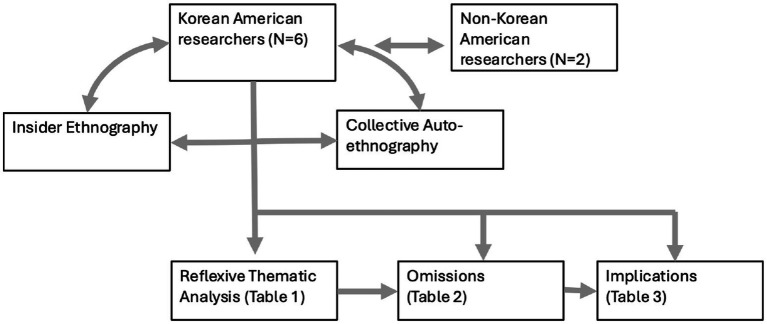
Dialectic between Korean American researchers and Korean American participants to understand racial and ethnic discrimination.

However, we acknowledge that the Korean American experience is multifaceted, and we cannot provide insights that are representative of the entire heterogenous Korean American community. Moreover, we are researchers in academia, and therefore bring a specific view to the subject matter shaped by scholarly discourses surrounding racial discrimination and its impact on health outcomes in marginalized communities. However, our research team consists of members with intersectional experiences in terms of age, gender, immigration history, and acculturation. We reflexively discussed our positionality with two non-Korean American co-authors (one Filipina American, and one Singaporean American), who provided ‘outsider’ perspectives. Figure 2 shows the dialectic between participant and researcher in the analytic process, with participants driving the generation of themes, and with insider/auto-ethnography driving discussion around omissions and research implications.

### Analysis

Following the guidelines set forth by Braun and Clarke (2019), we used reflexive thematic analysis to code all transcripts, beginning with familiarization with the data, then generating codes, searching for themes, reviewing themes, and defining themes. Two of the authors reviewed and coded transcripts after each focus group, and inductively developed preliminary codes. After all four focus groups were conducted, we deliberated and identified the major themes. We then reviewed the codes and themes in group meetings, and used our own insights, observations, and lived experiences to make sense of focus group data. We also drew from our own lived experiences to identify omissions and articulate main implications for research.

## Results

After iterative rounds of coding and discussion, we established four key themes based on the focus groups, which we present in Table 1.

1 Racial discrimination is often vague, subtle, and unclear.

**Table 1:** Themes.

Themes	Notes
1. Racial discrimination is often vague, subtle, and unclear.	Racial discrimination was difficult to describe Some did not find discrimination (positive stereotypes) to be bothersome Some downplayed experiences of discrimination vis-à-vis the experiences of other racial groups
2. Ethnic discrimination targeting Korean Americans is not differentiated from racial discrimination against Asian Americans more broadly.	Some felt lumped into a monolithic Asian group and were mistaken for another ethnic group. Racial discrimination was thought of in terms of race, but pride was thought of in terms of ethnicity
3. Certain measures of racial discrimination against Asian Americans were perceived as inadequate.	The AARRSI measure had outdated cultural references
4. Historical events may have altered perspectives on racial and ethnic discrimination. a. Greater awareness of one’s minoritized status as Asian American and increased racial discrimination. b. Increased awareness of race and racism affecting people of color as a whole.	Changes in perspectives over time occurred within historical moments or contexts Some became more aware of the prevalence of racism impacting people of color as a whole Cultural pride was based on ethnicity, not race

Before the beginning of the COVID-19 pandemic and BLM, from the perspective of Korean American emerging adults, we found that some respondents felt that the racial discrimination that they experienced was vague. One respondent expressed difficulty characterizing the type of discrimination she experienced, stating that she “cannot really explain it” (female, 22). Another respondent stated, “I guess it’s more of an overall sense of it’s kind of like the indescribable. It’s not tangible right in a sense, but it’s like some that you can just feel” (male, 20).

Along these lines, the respondents perceived that racial discrimination was more subtle against Asian Americans, and more blatant against other racial groups. As one respondent put it:

I don't know about everyday life. But it is definitely something I noticed because obviously I understand that the Asian American discrimination isn't as great in magnitude as other racial, other races but because of that, I feel like a lot more things are being done to eliminate racism for them. …but for Asian Americans, I feel like because we're model minority, we're not as taken care of, I guess. I don't necessarily notice it every day… But when I sit down and reflect like I am now I'm like, there are so many instances where I was being subtly discriminated [against], I guess, versus directly which might happen more for other races and just because it's more subtle, it's more unaddressed (female, 19).

Here we saw that the respondent did not provide concrete examples of racial discrimination and rather described the experiences of Asian Americans to be less severe compared to the experiences of other racial groups. Another respondent stated that the racial discrimination against Asian Americans was not bothersome or “not too upsetting” [male, 20]. The male respondent went on to state:

Kind of going off of that. I think something that I noticed is, in general, among Korean Americans is being identified as model minority. …Well, I'm not really too offended by it, when I hear something like that, but it's… I feel like it kinda makes light of the struggle that Asian Americans kinda go through. … Oh, it's there, it's always kind of assumed. To be honest, I don't really get too upset (male, 20).

The respondent stated that being regarded as a model minority was only slightly bothersome, that it was “there, it’s in the back of my mind” but that he did not let it trouble him.

A complicating historical factor that may influence perceptions of racial and ethnic discrimination is the globalization of Korean popular culture, Korean Wave (known as Hallyu), specifically the K-pop band BTS, and Korean films such as *Parasite*. Respondents expressed positive sentiments about the globalization and representation of Korean culture.

I think in that sense, it's kind of changed. … Korean culture [is] kind of spreading, being so widespread, has kind of changed people's views …not just Western media but global media…. So I think that's a great, you know, a step in the right direction (male, 20).

Another respondent pointed out the trends could work as a connection between Korean Americans and other groups:

People who like Korean or K-pop are probably going to be more friendly toward Korean people because they feel like they have something in common, and that they feel like they have something that they can talk about more they feel like some they have something special that they both share. So I think definitely being able to have more Korean culture and popular media would be a really good thing. It would bring a lot more positive things than harm (male, 20).

However, one respondent stated that it can be slightly offensive for people to assume that all Korean Americans listen to BTS or watch K dramas, which may constitute a kind of stereotyping.

2 Ethnic discrimination targeting Korean Americans was not differentiated from racial discrimination against Asian Americans more broadly.

We found that in general participants did not reflect deeply on the differences between race and ethnicity in terms of discrimination. On one hand, the participants in our groups suggested that it was offensive to be mistaken for other Asian American ethnic groups. For instance, one respondent described the common experience of being lumped into the same category as other Asian Americans.

I think most of the time… I feel like Asian Americans aren't really seen as unique in terms of the American perspective. Everyone just assumes that we're all from… Cause we're all East Asians, so I don't really see it being differentiated but I personally know that it's different and stuff… A lot of Americans don't even acknowledge Asian American. Let alone specific [ethnicity] between Asian Americans (female, 19).

At the same time, we found that Korean American emerging adults were not able to clearly differentiate discrimination against Korean Americans specifically from discrimination against Asian Americans more broadly. When we asked respondents to list experiences of discrimination, respondents mentioned experiences that could be applicable to other Asian groups, such as being confused with other Asian ethnicities, being called Asian slurs, and having Asian stereotypes imposed upon them. Respondents would often describe discriminatory events but did not think it was unique to Korean Americans.

One respondent described the experience of being a ‘perpetual foreigner,’ a stereotype that members of ethnic minorities will perpetually be seen as the “other” in the White dominant society (Huynh et al., 2011), as an ongoing form of exclusion and marginalization. While describing her experience attending a predominantly White university, she stated:

There was this one experience at the scholarship meeting where… [an alumna] looked at me, she was like, "So when did you come to America?" … And I was like, "Oh I was born here, I was raised here." I felt like I had to explain myself… it's just for me it's very… A bit offensive to me (female, 22).

Here we saw the participant describing an experience of being treated as a foreigner, which is a common microaggression that Asian Americans experience (Huynh et al., 2011).

Being mistaken for another ethnic group appeared particularly offensive because respondents were proud to be Korean. One respondent stated:

… It was a very different experience from what I've been living my whole life, that it was weird that I had to explain myself that I am actually American. I have been born here and raised. All of them were like "What ethnicity are you? Are you Chinese?" Just the assumption that you’re Chinese is… I get offended a little bit, because I'm proud to be Korean and when people assume that you're a different race without actually asking at first, and just guessing, it's just for me it's very… A bit offensive to me (female, 22).

We saw that the offense of conflating all Asian ethnic groups together lies in the diminishment of one’s heritage, culture, and ancestry. That is, people may talk about discrimination in terms of race but may talk about pride in terms of ethnicity.

3 Certain measures of racial discrimination against Asian Americans were perceived to be inadequate.

Participants stated that the AARSSI measure seemed out of date. For example, one item on the scale referred to Asian American journalist Connie Chung. Participants asked: “Who is Connie Chung?” One respondent was able to reason that perhaps the item was intended to elicit experiences of being confused for other Asian Americans. “Kind of similar to that is ‘Oh my god, you look just like my other Asian American friend.’ That I get pretty frequently” (female, 19).

Another respondent pointed out that certain stereotypes appeared dated as well, such as the AARSSI item about knowing martial arts:

.. I feel like most people know now that Korean Americans have their own martial arts, Tae Kwon Do. And also, I don't think people assume that I do Tae Kwon Do. So I've never had anyone ask me if I know how to karate. Maybe it's because I'm a girl… (female, 19).

Here we see that the stereotypes are also gendered, which is generally not accounted for in existing measures of racial discrimination among Asian Americans.

In response to the item that “Asian characters in American TV shows either speak bad or heavily accented English” a respondent suggested an update to reflect the recent changes in media.

I would kind of modify it because now it's not speaking badly or heavily accented as much but it's more like the roles. They have other… It's like a submissive role or a complementary or supplementary role to a main white character, or like is not being represented in general. The fact that Crazy Rich Asians was the first all-Asian cast and in 25 years is absolutely ridiculous (female, 19)

4 Historical events may have altered perspectives on racial and ethnic discrimination

After the start of the COVID-19 pandemic and BLM protests, we conducted another focus group, inviting previous participants back for discussion about how their experiences may have changed. We found that historical events tended to modify their perceptions of racial and ethnic discrimination. We identified two key sub-themes.

4a. Greater awareness of one’s minoritized status as Asian American and increased racial discrimination.

In 2020, several historical events occurred that may have altered perspectives on racial/ethnic discrimination. COVID-19 pandemic spread across the country, giving rise to anti-Asian sentiment, including a steep rise in hate crimes against Asian Americans. On June 20, 2020, the former President Trump, referred to COVID-19 as “Kung Flu.” Over 1,800 incidents of racism had been reported in the United States since the outbreak over an eight-week period (Borja and Gibson, 2020). Since the outbreak, studies estimated that over 40% reported that other people acted uncomfortably around them, 31% reported being called racial/ethnic slurs, jokes, and 26% feared being physically attacked (Ruiz et al., 2020). These events may have increased awareness of racial discrimination experiences and the significance of these experiences, raised awareness of the racial discrimination that other people faced, and how these events can serve as opportunities for connection and solidarity across ethno-racial groups.

We invited respondents to participate in a follow-up focus group and revisit their reflections on racial discrimination considering these events. Respondents appeared to exhibit a shift in perspective. One respondent (male, 20) offered a personal story of how the people in his family were affected by racial discrimination in this context and shared that his relatives had been verbally harassed on public transportation with comments blaming them for the COVID-19 pandemic.

Another respondent noted the increased racial discrimination.

“…we’re facing more discrimination… It just became a lot harder than it was before. And, I’ve been hearing that it’s not going to go away for a long time too … this kind of sentiment against Asians, especially, you know, East Asians, and you know, Chinese Americans, but you know a lot of Americans can’t tell East Asians apart…” (male, 20).

The respondent appeared to suggest that animus directed at Chinese Americans would invariably impact Korean Americans as ‘collateral damage’ since people may place all Asian ethnic groups in the same monolithic category. Considering the hostile climate toward Asian Americans, respondents suggested a greater awareness of their own racial identities:

Get food or get groceries or something… I felt a little more conscious of [my] race than I normally would have and I feel like a lot of Asian Americans probably can agree to that, to some extent…. (male, 20).

Some participants even stated that they use code-switching, intentionally speaking in English in front of others to minimize the perception that they are foreigners: “I can understand where some people might be coming from but yeah I think I’m definitely aware of it and … I try to speak as much, you know, English as I can so people do not think that way and then kind of come out aggressively (male, 20).

4b. Increased of race and racism affecting people of color as a whole.

Another historical moment that occurred in 2020 was the death of George Floyd at the hands of police and subsequent BLM protests that erupted across the country because of a longstanding problem of police violence against Black people. Reflecting on BLM, respondents shared the importance of minority groups’ supporting each other against racism.

I think minorities [should] kind of stick together and support one another because like, in the world we are minorities. So, you know, there's just not that many of us. So I think it's important … to be able to support the movement so that in the future when there's… a widespread movement like that for… like Asian Americans as well. Then, like our Black brothers and sisters can kind of support us as well. And we can kind of stand as like a united front. Take for kind of equality and things like that, especially in terms of kind of like systemic racism. … I just think that's something that really needs to change. And we really need to bring the light to not just for Black Americans but for you know Asian Americans as well. And I think when that time comes, and it'd be nice to have kind of united front and support one another (male, 20).

Echoing the above statement, another respondent distinguished the 2020 BLM movement from past movements as one supported and attended by Asian Americans and expressed hopes for future changes.

… This movement for Black Lives Matter for the 2020 … the one encouraging thing is it was so diverse, you know, the people attending these protests, it wasn't all just Black people. It was largely you know it was minorities. Asian people, White people too. …it's a hope and sign for gradual change in the future and which is like really encouraging (male, 20).

In short, historical events may have given rise to experiences of racial discrimination, both direct and indirect, and may have provided the context where individuals formed views of themselves and their places within society.

### Collective insider/auto-ethnographic reflections

After synthesizing our memos and meeting to discuss, we reflected on several forms of discrimination several forms of discrimination that were not raised in focus groups. These included online racism (e.g., harassment in virtual spaces), and appearance-based discrimination (e.g., body shaming and the privileging of Eurocentric beauty standards). We noted that respondents did not describe romantic and dating biases, including racialized sexualization, fetishization, and emasculation. We recognized that it is possible some types that it is possible some types of discrimination were not reported due to life stage. For example, participants might not have reported certain microaggressions (e.g., being overlooked, interrupted, or under-recognized in professional contexts) because they were still students. Other observations we noted included the omission of cultural racism, such as lack of media representation and stereotypes of Asian Americans as being one-dimensional (e.g., college admissions). We organized our observations in Table 2.

**Table 2:** Insider and auto-ethnographic reflections of types of discrimination that were not raised in the focus groups (*N*_r_ = 6).

Omissions	Examples
Online racism	Online harassment and prejudice encountered in virtual spaces.
Appearance-based discrimination	Body shaming and being considered undesirable due to facial features or body types/parts. Exaltation of Eurocentric beauty standards
Romance/dating	Online dating sites and applications. Lotus blossom or dragon lady stereotypes. Sexualization, fetishization, and emasculation. Dating behaviors
Age-related microaggressions	Being perceived as young and not taken seriously
Power dynamics and microaggressions related to attention or recognition	Experiences of being pushed around, taken advantage of, or spoken over in professional or social settings. Being overlooked for promotions or leadership roles Not being properly acknowledged or being under-rewarded at work Expectations of being docile or demure
Tokenism	Feeling like an obligatory presence to fulfill diversity goals rather than being valued for individuality.
The burden of positive stereotypes	The pressure to live up to unrealistic expectations associated with the model minority myth
Intersectionality	Feelings of being rejected or devalued in both White and heteronormative spaces Exposure to racial discrimination across socioeconomic status. Forbidden from having intersectional identities. Not being allowed to be both Korean and American (e.g., Which country do you root for in the Olympics?).
Cultural racism	Lack of representation in media Conversations about affirmative action and stereotypes that portray Asian Americans as robotic, one-dimensional students who lack cultural depth or social skills. Stereotype threats Situated in a White-dominant society without “counting” toward diversity, especially in educational settings.
Comparisons	Being pitted against other racial/ethnic minorities Perceived by other racial/ethnic minorities as adjacent to White people (and somehow beneficiaries of White supremacy) and viewed by White people as minorities that support the narrative of meritocracy (if you work hard you will be rewarded), which can ultimately be used against other racial/ethnic minorities.

While we did not delve into the complexities of racial and ethnic identity with the focus group participants, we did allow space for them to reflect on the difference between race and ethnicity. We noted that the examples of discrimination they reported were largely applicable to other Asian racial groups (not unique to Korean Americans), including the experience that people think all Asians are the same. However, when asked about their Korean identity, participants pointed to ethnicity-specific examples of culture and pride. A thought-provoking scenario may be to consider which countries Korean Americans root for during the Olympic Games; Korean Americans may be proud of both South Korea and the US, and may feel somewhat divided when the countries are competing against each other. But Korean Americans may not often feel any particular sense of pride for other East Asian countries, such as China or Japan.

## Discussion

The purpose of this study was to investigate how Korean American emerging adults in higher education perceived racial and ethnic discrimination, and to examine any changes in perspectives in light of socio-historical events, specifically the COVID-19 pandemic and BLM. Our study revealed nuanced findings about how Korean American emerging adults viewed racial discrimination. We found that Korean American emerging adults saw racial discrimination as vague, subtle, and unclear; that ethnic discrimination targeting Korean Americans was difficult to differentiate from racial discrimination against Asian Americans more broadly; that certain measures of racial discrimination against Asian Americans seemed inadequate; and that historical events may have altered perspectives on racial and ethnic discrimination. We discuss these themes below.

### Racial discrimination is often vague, subtle, and unclear

Existing literature has underscored the covert or invisible nature of racism (Bonilla-Silva, 2006; Noh et al., 2007; Jones et al., 2016; Jadotte et al., 2022), and so it is not surprising that the respondents had difficulty describing their experiences of racial discrimination. Some respondents felt that they were not seriously impacted by racial discrimination, pointing to positive stereotypes such as the model minority myth, which did not feel particularly bothersome. Also, the ongoing ‘Korean Wave’ and globalization of Korean popular culture often seemed to give an impression that Korean Americans were embraced in American society. These views coincide with emerging literature that has explored why ethno-racial minorities may deny or minimize racial discrimination (Kay et al., 2013; Oh et al., 2025; Siy and Cheryan, 2016; Yi et al., 2022). Notably, ethno-racial minorities may feel as though their experiences do not ‘count’ when compared with more serious forms of racism (Lewis et al., 2015; Oh et al., 2025), such that Korean Americans may minimize their own struggles relative to the more visible and often violent forms of racial discrimination faced by Black communities (e.g., police brutality). What some may consider subtle discrimination, however, can be more psychologically and physiologically challenging than blatant experiences of discrimination (Noh et al., 2007; Yoo and Lee, 2008). The findings highlight the complexities in perceived racial discrimination experiences and warrant a careful examination of how vague and subtle experiences may affect well-being.

### Ethnic discrimination targeting Korean Americans is difficult to differentiate from racial discrimination against Asian Americans more broadly

We discovered that respondents could not clearly separate ethnic discrimination targeting Korean Americans from broader anti-Asian American racial discrimination, making it unclear if Korean Americans faced unique forms of discrimination compared to other Asian American subgroups. Thus, we did not find compelling evidence that an ethnic-specific measure of discrimination was warranted. Existing measures of anti-Asian discrimination already address experiences such as feeling like a ‘perpetual foreigner’ (the enduring perception that Asian Americans are outsiders and do not truly belong in the USA, regardless of whether they were born in the country or how long they have lived in the country (Liang et al., 2004; Yoo et al., 2010; Huynh et al., 2011), which resonated with the Korean American emerging adults in our focus groups.

At the same time, participants expressed frustration at being conflated with other Asian Americans, which was a common kind of discrimination described in the focus groups (i.e., all Asians are the same). One study of Korean American high school students found that most of the sample identified solely as Korean, rejecting a pan-ethnic Asian identity as Asian, and even working hard to distinguish themselves from other Asian Americans (Lee, 2009). It seemed that when discussing experiences of racial discrimination, respondents tended to point to race; however, when discussing aspects of identity, like cultural pride, respondents tended to refer to ethnicity. For instance, the Korean focus group participants mention the popularization of K-Pop, K-Drama, and K-food as a source of Korean pride. And yet when describing experiences of discrimination, the participants tended to describe stereotypes that were common to other Asian American groups.

### Certain measures of racial discrimination against Asian Americans seem inadequate

A clear finding of our focus groups was that measures of anti-Asian racial discrimination can be inadequate. When presented with AARRSI, respondents were unfamiliar with the cultural references, underscoring the idea that racial discrimination scales are time-bound and reflect the specific forms of prejudice prevalent when the scales were developed (see Hester et al., 2023). Upon reflecting on the participants’ experiences and their reactions to the AARSI, we drew from our own observations and experiences to highlight the kinds of discrimination that were not discussed in the focus groups (Table 2). We offer these omissions to complement the focus groups findings, given that the items from the AARSSI did not touch on several key domains where Korean Americans may experience racial discrimination.

### Historical events may have altered perspectives on racial and ethnic discrimination

Our focus groups offered a unique opportunity for us to explore how the COVID-19 pandemic and BLM protests may have influenced Korean American emerging adults’ perceptions of racial discrimination. The findings suggest that historical events, particularly ones blatantly revealing racism in our society in our society, contextualize and shape participants’ perceived racial discrimination experiences. Prior to the COVID-19 pandemic, participants described experiencing racial discrimination infrequently, being uncertain about whether they had experienced racial discrimination, or not being bothered by the experiences. The COVID-19 pandemic increased direct and indirect exposure to racial discrimination, and Korean Americans may have personally experienced increased discrimination due to their ethnicity (being conflated with other Asian Americans) or witnessed media coverage of racial discrimination against other Asian Americans (Yu et al., 2020; Layug et al., 2022), increasing awareness of group-level discrimination, racial biases, and the public’s low regard for Asian Americans (McGarity-Palmer et al., 2023).

Our findings align with other qualitative work on Asian Americans during the COVID-19 pandemic. Lee et al. (2023) found that Asian American women reported experiencing a range of COVID-19–related racial discrimination, including direct verbal harassment and slurs, as well as avoidance and exclusionary behaviors where people intentionally distanced themselves. Participants also described being stereotyped and blamed for spreading the virus, and being ‘othered’ or treated as perpetual foreigners. In addition to these direct encounters, many experienced internal or vicarious discrimination by worrying about family members’ safety or hearing about others’ racist experiences. These incidents had a range of emotional and psychological consequences (e.g., anxiety, fear, hypervigilance, sadness, anger, internalized self-blame) and behavioral consequences (e.g., social isolation, modifying physical appearance, or avoiding places) to avoid discrimination. An important aspect of their study is that the experiences prompted deep reflection on racial identity, including heightened awareness of being Asian, prompting negotiation between assimilation and pride, and shaping self-presentation. This aligns closely with our findings.

The second precipitating event was the BLM protests, which may have prompted Korean Americans to reflect on their own minority status vis-à-vis the broader struggles against racial injustice faced by Black communities. Korean Americans may have become more aware of racism and may have come to realize that they too experience racial discrimination in a racist society (Matriano et al., 2021). On the other hand, it is also possible that Korean Americans questioned whether their experiences whether their experiences counted as racial discrimination compared with the experiences of Black Americans. This ‘minority comparison effect’ has been documented in emerging literature (Baek and Lee, 2012; Oh et al., 2025). Both COVID-19 related anti-Asian discrimination and the BLM protests may have led Korean Americans to re-evaluate their perceptions of racial discrimination and their roles in fighting racism.

### Implications

We generated a list of implications for future research (Table 3), which should be viewed next to the larger body of work on racial discrimination measurement (Atkins, 2014; Lewis et al., 2015). Racial discrimination is often experienced as vague, subtle, and ambiguous, underscoring the need for culturally sensitive measurement, such as clarifying terms through dialogue boxes, providing concrete examples, probing for minimization, and including prompts about multiple domains like online spaces. We also noted that ethnic discrimination targeting Korean Americans can be difficult to distinguish from broader racial discrimination, suggesting that researchers should ask about both race and ethnicity, anticipate confusion around these concepts, and attend to ethnicity-specific experiences that vary across subgroups. For example, South Asians may be more likely to report being racially profiled at security in airports whereas East Asians might be stereotyped for knowing martial arts or ridiculed for eating certain types of foods. Further, existing racial discrimination measures may be inadequate, requiring updated cultural references, attention to life stage, gender, and sexuality, and the creative integration (“curation” or “Frankensteining”) of items across scales to capture nuanced experiences. Finally, historical events may shape perceptions and reporting of discrimination, highlighting the value of context-sensitive approaches such as case studies, process tracing, and the use of visual or media prompts to evoke reflection on evolving social realities. Our work also encourages the integration of qualitative approaches into longitudinal designs, where quantitative frequency of racial discrimination measures before and after historical events may help strengthen causal inferences, while qualitative inquiries can triangulate and add depth to perceptions of change over time.

**Table 3:** Potential implications for research derived from insider and auto-ethnographic reflections of focus group content (*N*_r_ = 6).

Themes	Potential implications for research
Racial discrimination is often vague, subtle, and unclear	When possible, administer racial discrimination measures to multiple Asian ethnic groups and explore cultural equivalence across groups Use dialogue boxes to clarify terms Use prompts to cue the respondent to reflect on multiple domains where discrimination may occur, including online spaces Offer concrete and timely examples of racial vs. ethnic discrimination in parentheses Probe when denial or minimization occurs Clarify experiences in instructions and question stems
Ethnic discrimination targeting Korean Americans is difficult to differentiate from racial discrimination against Asian Americans more broadly.	Ask about both race and ethnicity, but anticipate that people may not have reflected deeply on the differences between race and ethnicity or be confused by the terms Discrimination may be experienced in terms of race, but certain aspects of identity such as pride may be more closely understood in relation to ethnicity. Interview for ethnicity-specific experiences Acknowledge that different ethnic groups may experience different types of discrimination. For example, South Asians may report higher frequencies of racial profiling at security checkpoints and police encounters when compared with East Asians.
Certain measures of racial discrimination against Asian Americans seem inadequate.	Use updated cultural references Account for life stage; for emerging adults, consider that some discrimination is a function of age and socioeconomic status (e.g., one cannot endorse being overlooked for promotion if one is still in college and not in the labor market; one cannot be denied a bank loan if one is not in a position to purchase a home in the first place) Account for genders and sexualities Provide examples of racial discrimination in parentheses Adopt an eclectic approach to measurement and explore whether it is feasible to use multiple discrimination measures Explore potential ways to ‘mix and match’ items or subscales to capture a fuller picture of Korean Americans. Here we offer terms such as ‘curate’, ‘cobbling’, or ‘Frankensteining’ items and subscales together into a measure.
Historical events may have altered perspectives on racial and ethnic discrimination	Researcher should be attuned to historical moments in which the measures were created and administered Case studies, process tracing, and other methods can elucidate how historic events lead to changes in reporting behaviors Use photos, headlines, and other media to prompt/guide reflection

### Limitations

As with all studies, our study should be interpreted considering its limitations. One potential limitation is that the focus groups were initially held in person but converted to the Zoom platforms. It is unclear how this may have changed the group dynamics among the participants. Additionally, convening a follow-up focus group with a different configuration of members who were available does not capture a complete picture of how respondents changed their views over time although at least one participant from each focus group was represented in the follow-up. In terms of sampling, the study consisted of an urban, English-speaking, college educated emerging adults. There may be considerable within-group variation in terms of perceptions of racial and ethnic discrimination within Korean communities (e.g., differences based on political ideology, acculturation, immigration status, sexual orientation), which might not be adequately captured in the current study. Finally, as insider- and auto-ethnographers, we must acknowledge our multiple roles as both Korean Americans and as researchers, and the vantage points from which we are viewing the complex sociocultural phenomena across a heterogeneous community. We reflexively embrace our identities as researchers and understand the limitations of our roles.

## Conclusion

In conclusion, Korean American emerging adults reported that racial discrimination they experienced tended to be vague, subtle, and nuanced, and therefore difficult to describe. However, historical events, such as the COVID pandemic with heightened negative sentiments toward Asian Americans drastically increased more blatant forms of racial discrimination. Witnessing struggles and injustice toward another racial/ethnic minority community (i.e., police violence toward Black communities) may have triggered a reflective process among Korean American emerging adults, which may have led them to reflect on struggles across communities of color in a society where racism is deeply rooted. Respondents viewed some aspects of a particular racial discrimination measure to be inadequate. Although frustration of being conflated with other Asian Americans and being stereotyped has been reported, an Asian-ethnic group specific measure of racial discrimination has not been supported. Respondents tended to report discrimination in terms of race but tended to report aspects of identity and pride in terms of ethnicity, suggesting that Asian ethnic group specific identity measures should be considered. Future research may explore ways to improve discrimination measures for Asian Americans by specifying and asking more explicitly about experiences; clarifying experiences and probing more deeply into experiences; asking about both race and ethnicity; acknowledging historical timing and location; and to bringing together different measures, subscales, or items.

## Data Availability

The datasets presented in this article are not readily available because NA. Requests to access the datasets should be directed to hansoh@usc.edu.
