# Supplementary material for: Ethnic-specific discrimination: focus group findings of Korean American emerging adults
Source: Front Sociol. 2026 Jan 7;10:1658624. doi: 10.3389/fsoc.2025.1658624 (PMC12819650; doi:10.3389/fsoc.2025.1658624)
Supplement: Supplementary file 1 [file Table_1.docx]

**SUPPLEMENTAL MATERIALS**

**Table S1A. Characteristics of focus groups**

| **Focus group** | **N** | **Gender** |
| --- | --- | --- |
| Group 1. September 2019 | 4 | 3 women, 1 man |
| Group 2. October 2019 | 4 | 1 woman, 3 men |
| Group 3. March 2020 | 5 | 2 women, 3 men |
| Group 4. December 2020 | 4 | 1 woman from group 1; 2 men from group 2; and 1 man from group 3 |
| Self-reported gender identities. | | |

**Table S1B. Characteristics of focus groups**

| **Participant** | **Focus group** | **Gender** | **Age** | **Religion** | **Years in U.S.** |
| --- | --- | --- | --- | --- | --- |
| 1 | 1, 4 | Female | 22 | Christian | 22 |
| 2 | 1 | Female | 19 | Catholic | 19 |
| 3 | 1 | Female | 19 | Christian | 16 |
| 4 | 1 | Male | 20 | Christian | 7.5 |
| 5 | 2, 4 | Male | 20 | Christian | 20 |
| 6 | 2 | Male | 24 | Christian | 15 |
| 7 | 2, 4 | Male | 19 | N/A | 17 |
| 8 | 2 | Female | 19 | N/A | 14 |
| 9 | 3 | Male | 22 | Christian | 7 |
| 10 | 3 | Male | 20 | Agnostic | 20 |
| 11 | 3, 4 | Male | 20 | Catholic | 20 |
| 12 | 3 | Female | 18 | Christian | 18 |
| 13 | 3 | Female | 21 | Christian | 21 |

| **Table S2. Examples of Interview Questions** |
| --- |
| - When was the first time that you experienced or witnessed racial discrimination or microaggression? - How has discrimination impacted your life? - Do you think you experience discrimination more these days as a young adult, in comparison to when you were a child? - What types of racial discrimination have you witnessed that your parents experienced? - Do you think that Korean girls and boys experience similar types of racial discrimination/microaggression? - We would like to know your thoughts about the AARSSI scale. Please rate the items in terms of relevance, distress, and frequency. |
